# Preferential Transfer of Certain Plasma Membrane Proteins onto T and B Cells by Trogocytosis

**DOI:** 10.1371/journal.pone.0008716

**Published:** 2010-01-14

**Authors:** Sandrine Daubeuf, Anne Aucher, Christine Bordier, Audrey Salles, Laurent Serre, Gérald Gaibelet, Jean-Charles Faye, Gilles Favre, Etienne Joly, Denis Hudrisier

**Affiliations:** 1 CNRS, IPBS (Institut de Pharmacologie et de Biologie Structurale), Toulouse, France; 2 Université de Toulouse, UPS, IPBS, Toulouse, France; 3 Centre d'Immunologie, Marseille Luminy, France; 4 INSERM U 563, Hôpital Purpan, BP 3048, Toulouse, France; 5 INSERM U 563, Institut Claudius Régaud, Toulouse, France; University of Nebraska Medical Center, United States of America

## Abstract

T and B cells capture antigens via membrane fragments of antigen presenting cells (APC) in a process termed trogocytosis. Whether (and how) a preferential transfer of some APC components occurs during trogocytosis is still largely unknown. We analyzed the transfer onto murine T and B cells of a large panel of fluorescent proteins with different intra-cellular localizations in the APC or various types of anchors in the plasma membrane (PM). Only the latter were transferred by trogocytosis, albeit with different efficiencies. Unexpectedly, proteins anchored to the PM's cytoplasmic face, or recruited to it via interaction with phosphinositides, were more efficiently transferred than those facing the outside of the cell. For proteins spanning the PM's whole width, transfer efficiency was found to vary quite substantially, with tetraspanins, CD4 and FcRγ found among the most efficiently transferred proteins. We exploited our findings to set immunodiagnostic assays based on the capture of preferentially transferred components onto T or B cells. The preferential transfer documented here should prove useful in deciphering the cellular structures involved in trogocytosis.

## Introduction

Over the past few years, the intercellular exchange of PM proteins between cells of the immune system has been reported many times [1], [2]. Different terms have been used to describe this process such as trogocytosis [2], shaving reaction [3], nibbling [4], swapping [5] or snatching [6]. It is still unclear if these different terms describe similar or different phenomena. The mechanisms whereby membrane fragments can exchange between cells are still largely unknown but it appears that not all cellular components can be exchanged. On the one hand, using fluorescent chemical probes targeting various subcellular compartments, we and others reported that dyes located at the PM rather than intracellular ones were transferred, suggesting some selectivity based on the location of labels within the cell [7], [8]. Regarding selectivity within the PM, however, using global labels such as lipids, proteins or glycoconjugates, we could not document selectivity of transfer since all these components were efficiently captured [7], [9]. On the other hand, Western blotting of biotinylated proteins captured by T or NK showed that only a subset of the PM proteins expressed by target cells were concerned, suggesting that some selectivity does occur, at least amongst the proteins present at the PM [8], [10]. Furthermore, using antibodies against candidate surface proteins, it was found that only some of them were transferred [6], [11].

Several vectors of intercellular communication that could account for the processes described above have been proposed [1], [5], [12], [13]. These include the formation of membrane bridges [14], membrane nanotubes [15]–[17], the secretion of vesicles including exosomes [18]–[22] or the tearing of membrane fragments [23]. Unfortunately, since there are no means to selectively block these processes, it is still impossible to unambiguously know which one(s) is (are) involved in the specific capture of membrane fragments by trogocytosis.

In the absence of specific ways to block the formation of the above structures, we reasoned that identification of proteins transferred or not during trogocytosis (to be compared with what is currently known on the identity of molecules conveyed by exosome/microvesicles [24], [25] or by nanotubes [26] for instance) could help deciphering the mechanism(s) of trogocytosis.

The exchange of PM components has been exploited as the basis for TRAP (TRogocytosis Analysis Protocol) assays as immunodiagnostic tools. Indeed, we and others have shown that the capacity of antigen-reactive T or B cells to capture PM components could be exploited to identify those lymphocytes within a complex mixture of cells [7], [9], [27]–[30]. TRAP assays have been based on the fluorescent detection of captured proteins [7], [9], [27], lipids [7], [9], [29] or glycoconjugates [9], or even of GFP-tagged MHC class I molecule [30], but this latter approach, which would require the generation of individual GFP-tagged versions for every single MHC molecule being studied, would be rather difficult to generalize. Identification of one or more GFP-tagged protein that gets efficiently transferred as a bystander during trogocytosis could, in principle, present several advantages for the detection of lymphocytes compared to biochemical components incorporated exogenously.

Therefore, we investigated the selectivity of the transfer at the protein level with the hope that the pattern of protein transferred (or not transferred) could provide us with clues on the mechanisms involved in trogocytosis and with the subsidiary aim of contributing to the development of TRAP assays. To this end, we undertook the analysis of the efficiency of transfer to murine T or B cells by trogocytosis of a number of proteins fused to auto-fluorescent proteins (AFP). We thus used a panel of AFP-fused proteins that target distinct subcellular locations such as lysosomes, endoplasmic reticulum, nucleus, cytosol, and the PM. We used a panel of PM proteins with various modes of insertion such as anchoring to the internal or external leaflet or integral membrane proteins, as well as various constructs expected to be enriched or excluded from membrane microdomains, the so-called «rafts». We found that only proteins present at the PM were detectably transferred, and evidenced some degree of selectivity in the transfer of certain PM-associated proteins during trogocytosis. Furthermore, we found that the identification of efficiently transferred proteins could be exploited for the design of novel immunomonitoring tools.

## Results

### Setup of an experimental system to evaluate the preferential transfer of fluorescent proteins by trogocytosis

**Figure 1:**
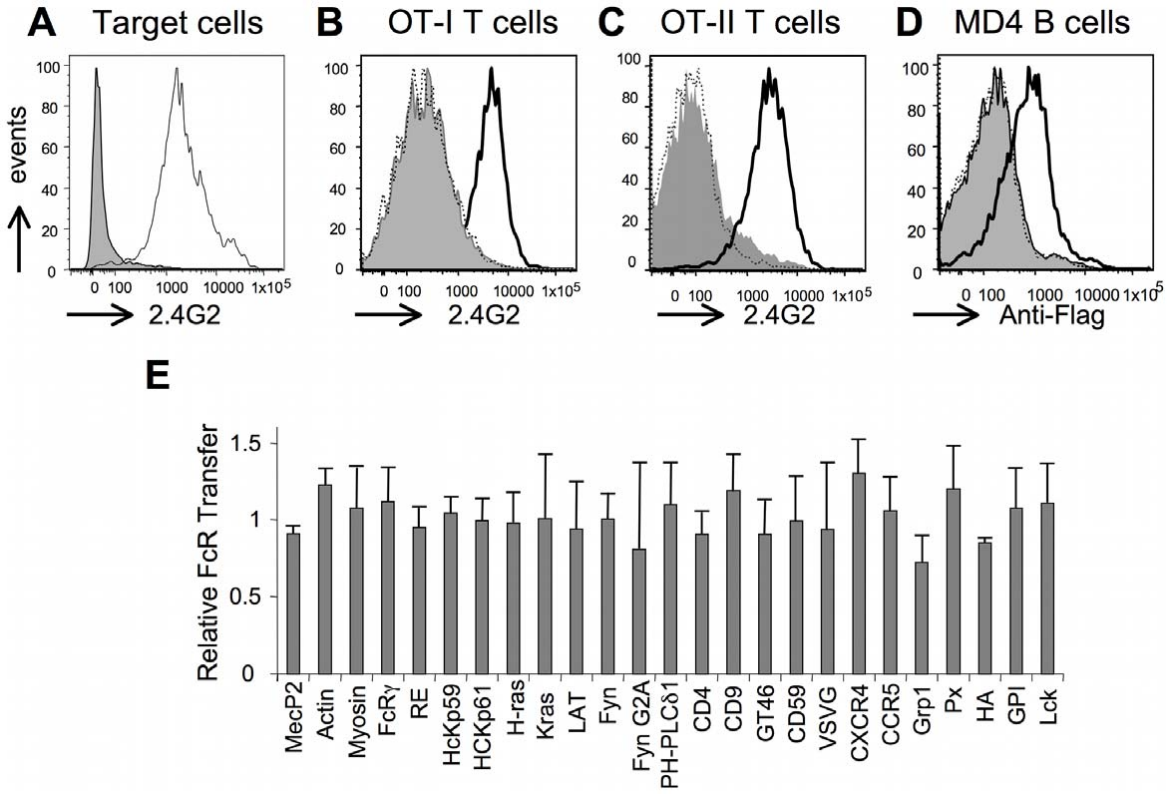
HEK-FcγRII cells and their transient transfectants are targets of murine T and B cells in redirected trogocytosis experiments. A) HEK cells (grey histogram) or HEK-FcγRII (white histogram) were stained with the anti-murine FcγRII/RIII mAb 2.4G2 and analyzed by flow cytometry. B) HEK-FcγRII cells were incubated 1 hour at 37°C with activated OT-I T cells in the presence (white histogram) or absence (grey histogram) of the Y3 mAb triggering trogocytosis or in the presence of its isotype control (dashed line) before analysis by flow cytometry using anti-CD8 and anti-FcγRII/RIII mAb. Shown are histograms of FcγRII/RII staining on gated CD8^+^ T cells. C) as in B) except that OT-II T cells were used instead of OT-I cells and were detected using anti-CD4 mAb. D) As in B) except that MD4 B cells coated or not with anti-κ chain mAb or its isotype control were used instead of OT-I T cells and that FcγRII/RIII capture was detected with the anti-Flag Ab on gated B220^+^ B cells. E) As in B) except that capture of FcγRII was analyzed after co-culture of OT-I T cells with HEK-FcγRII that were transiently transfected 48 hours earlier with vectors encoding the indicated proteins fused to GFP. Capture of FcγRII from HEK-FcγRII transfected with the indicated constructs is shown as fold induction, which was calculated as indicated in the Materials and Methods section and normalized with fold induction obtained in cocultures with untransfected HEK-FcγRII. Represented are means plus standard deviation from 3 independent experiments (no statistically significant differences emerged from student t-test analysis).

**Figure 2:**
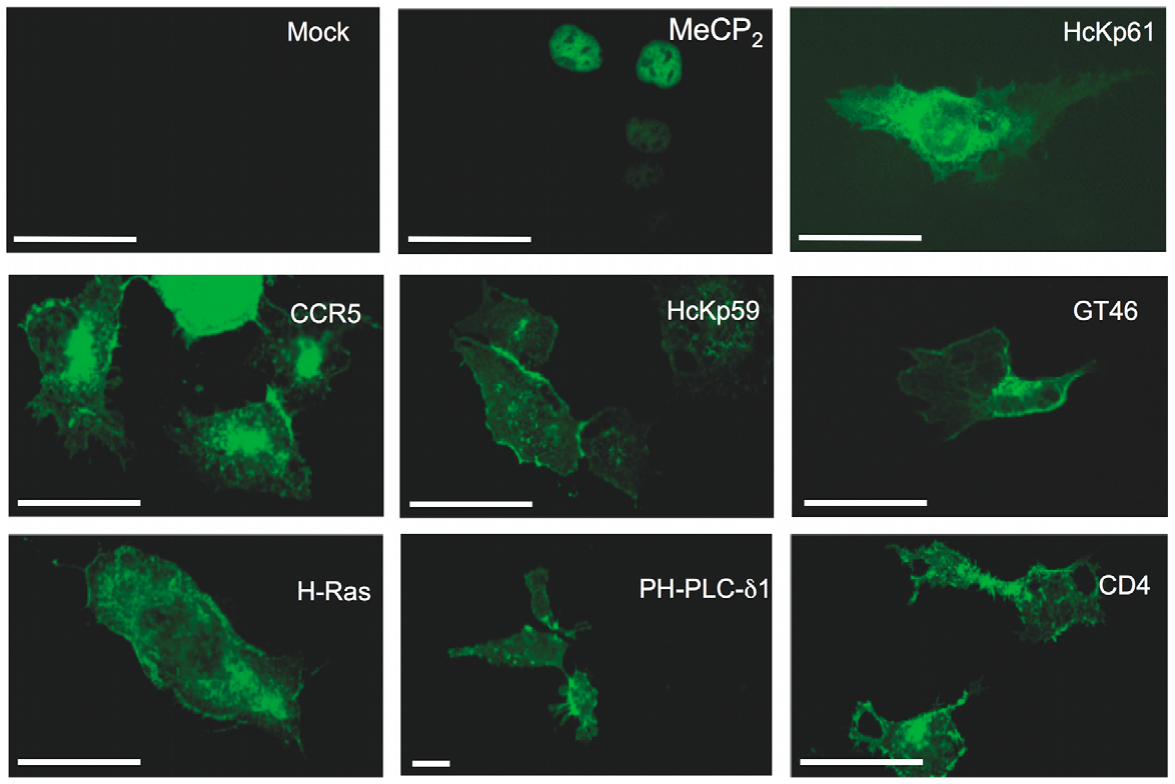
Localisation of GFP-tagged proteins overexpressed in HEK-FcγRII cells. HEK-FcγRII cells transiently transfected 48 hours earlier with constructs encoding the indicated GFP-tagged proteins were imaged by fluorescence microscopy. Although only nine transfectants are shown here for reasons of space, the localisation of each one of the proteins tested in this study was analyzed and found to conform to the localisation reported in the literature (see Table 1 and 2). Scale bars represent 50 µm.

With the aim of understanding if some proteins were more efficiently transferred than others during trogocytosis, we chose an approach whereby fluorescent forms of proteins representative of various subcellular localizations and various anchoring to the plasma membrane were expressed in transiently transfected cells. We would then co-culture these transiently transfected cells with either T or B cells, and analyse the efficiency of transfer of the GFP-tagged proteins by trogocytosis. Because only a small percentage of the components present on the target cells are captured by effector cells, we needed a system where the fluorescent proteins could be expressed at high levels and in a large proportion of target cells. Transient transfection of HEK cells is one of the more efficient and versatile systems for the expression of plasmid-encoded recombinant proteins in a mammalian cell. Therefore, we used HEK cells engineered to express a flagged form of FcγRII [31] (Figure 1A), which allows them to be targeted by T or B cells coated with appropriate antibodies (an approach termed «redirected trogocytosis”) [32], [33]. For OT-I CD8^+^ or OT-II CD4^+^ T, trogocytosis on the HEK-FcγRII cells was triggered very efficiently by the Y3 anti-H-2K^b^ mAb, as shown by the capture of the FcγRII receptor but not in the absence of mAb nor in the presence of a mouse IgG2a isotype control (Figure 1B and C). The capture of FcγRII receptor by T cells was simply detected with the 2.4.G2 anti-FcγRII/III mAb since T cells do not express these receptors endogenously. For MD4 B cells, we found that HEK-FcγRII cells could also be used as targets for redirected trogocytosis when the co-culture was performed in the presence of the anti-BCR κ chain mAb but not in the absence of mAb nor in the presence of the rat IgG1 isotype control (Figure 1D). In B cells, which constitutively express FcγRII, we used the Flag epitope carried by the recombinant FcγRII protein expressed by HEK cells to monitor trogocytosis with anti-Flag antibodies. Since, as in previous studies [32], we could not detect any differences (i.e. no trogocytosis was triggered) when isotypic controls were used as compared with no mAb added (Figure 1B–D) we chose to use controls simply performed in the absence of mAb for the bulk of our experiments. We obtained or generated a series of 32 different constructs encoding proteins fused to GFP or chromatic variants. The identity, source and expected subcellular location are summarized in Tables 1 and 2. When each one of these constructs was transiently transfected in HEK-FcγRII, a high proportion (>40%) of the cells consistently over-expressed the protein (see Figure S1). The vast majority of these constructs which encode proteins fused to AFP have been described before and extensively used by various laboratories worldwide (see reference for each of them in Table 1 and 2). For each of these constructs, we thus simply confirmed the expected localization of each fluorescent protein by fluorescence microscopy (Figure 2 and data not shown). For the few constructs generated in our laboratory, a more detailed analysis of their localization was performed using co-localization experiments with plasma membrane or nucleus markers (not shown). Furthermore, we confirmed the correct topology of the various fusion proteins using a polyclonal anti-GFP antibody in flow cytometry experiments, by checking that permeabilization was required in order to detect AFP moieties located intracellularly whereas this step was not necessary when the AFP moiety was accessible extracellularly (not shown). Importantly, we found no marked alteration in the overall efficiency of trogocytosis by OT-I CTL for any of the plasmids used, as detected by the capture of FcγRII (Figure 1E), indicating that the overexpression of these proteins does not detectably alter the interaction between target cells and effector cells. Similar results were obtained with OT-II cells or MD4 B cells (not shown). Thus, using redirected trogocytosis, HEK-FcγRII cells provide a versatile cellular system to study the capture of overexpressed, fluorescent proteins by T or B cells and the capture of FcγRII can be used to control for trogocytosis efficiency.

**Table 1:** Summary of the characteristics of the fluorescent proteins initially used in this study.

Protein	Cellular location	Type of anchor	Origin	Transfer to CD8^+^ T cells	Transfer to CD4^+^ T cells	Transfer to B cells
RE-GFP	RE	-	[61]	−	−	−
NLS-GFP	Nucleus	-	[61]	−	−	−
MeCP2-GFP	Nucleus	-	This study	−	−	−
CFP-Grp1	Nucleus	-	[62]	−	−	−
Actine-GFP	Cytosol	-	Clontech	−	−	−
Myosin-GFP	Cytosol	-	Addgene	−	−	−
GFP-PX	Endosomes	-	[62]	−	−	−
GFP-CD59	PMe	GPI	[63]	+	+	+
GFP-H-Ras	PMi+Golgi	Farnesyl, palmitoyl	[64]	++	++	++
GFP-K-Ras	PMi+Golgi	Farnesyl, poly basic domain	[64]	++	++	++
Fyn-GFP	PMi	Myristoyl, palmitoyl	[64]	+	+	+
VSVG-GFP	PMt	Transmembrane peptide	[65]	+	+	+
LAT-GFP	PMi	Transmembrane peptide	[66]	+	+	+
HA-GFP	PMt	Transmembrane peptide	[40]	+	+	+
YFP-GT46	PMt	Transmembrane peptide	[67]	+	+	+
CD4-GFP	PMt	Transmembrane peptide	[68]	++	++	++
CCR5-GFP	PMt	Transmembrane peptide	[68]	+	+	+
CXCR4-GFP	PMt	Transmembrane peptide	[68]	+	+	+
GFP-CD9	PMt	Transmembrane peptide	[69]	++	++	++
CD81-GFP	PMt	Transmembrane peptide	[70]	++	++	++
YFP-CD82	PMt	Transmembrane peptide	[71]	++	++	++
GFP-PH-PLC-δ1	PMa	-	[72]	++	++	++
P59HcK-GFP	PMi	Myristoyl, palmitoyl	[49]	+	+	+
P61HcK-GFP	Lysosomes	palmitoyl	[49]	−	−	−
GAP43-GFP	PMi	Palmitoyl, polybasic domains	[37]	+	+	+
FcRγ-GFP	PMt	Transmembrane peptide	[33]	++	++	++

PMe, external leaflet of PM; PMi, internal leaflet of PM; PMt, Spanning the PM; PMa, associated to the PM through interaction with phosphoinositide. Note that proteins were arbitrarily classified as not transferred (−, fold induction around 0.8–1.2), poorly transferred (+, fold induction in the range (1.5–3) and efficiently transferred (++, fold induction >3).

**Table 2:** Impact of PM anchors on the transfer efficiency of fluorescent proteins.

Protein	Cellular location	Origin	Transfer to CD8^+^ T cells	Transfer to CD4^+^ T cells	Transfer to B cells
***GPI-anchored proteins***					
GFP-CD59 (WT)	PMe	[63]	+	+	+
GFP-GPI (anchor only)	PMe	[63]	+	+	+
***Ras-family members***					
GFP-H-Ras (WT)	PMi+Golgi	[64]	++	++	++
GFP-CAAX-H-Ras (anchor only)	PMi+Golgi	This study; based on [64]	++	++	++
GFP-K-Ras (WT)	PMi+Golgi	[64]	++	++	++
GFP-CAAX-K-Ras (anchor only)	PMi+Golgi	This study; based on [64]	++	++	++
***Src-family members***					
Fyn-GFP (WT)	PMi	[64]	+	+	+
Fyn anchor GFP (anchor only)	PMi	[37]	+	+	+
Fyn anchor G2A-GFP (mutated anchor only)	Cytosol	[37]	−	−	−
P56Lckanchor-GFP (related anchor only)	PMi	[37]	+	+	+
Yes anchor-GFP (related anchor only)	PM	[37]	+	+	+

PMe, external leaflet of PM; PMi, internal leaflet of PM; PMt, Spanning the PM; PMa, associated to the PM through interaction with phosphoinositide. Note that proteins were arbitrarily classified as not transferred (−, fold induction around 0.8–1.2), poorly transferred (+, fold induction in the range (1.5–3) and efficiently transferred (++, fold induction >3).

### Quantitative differences in the detection of the capture of various fluorescent proteins in redirected trogocytosis

**Figure 3:**
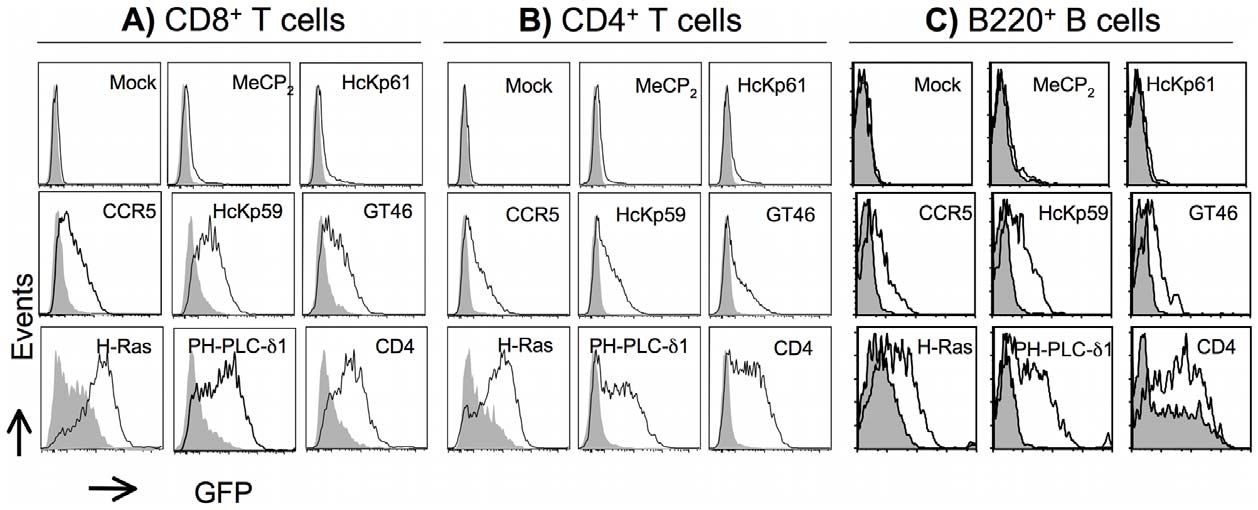
Quantitative differences in the detection of the capture of various fluorescent proteins in redirected trogocytosis. A) OT-I T cells were co cultured with HEK-FcγRII cells transiently expressing the indicated protein fused to GFP. Shown are histograms of GFP fluorescence on OT-I cells co cultured with the target cells in the presence (white histograms) or absence (grey histograms) of the Y3 mAb. Top, middle and bottom panels provide respectively three illustrative examples of proteins that are not significantly transferred, poorly transferred or efficiently transferred onto OT-I T cells during trogocytosis. B) As in A) except that OT-II T cells were used instead of OT-I T cells. C) As in A) except that MD4 B cells coated or not with an anti-κ chain mAb were used instead of OT-I T cells. The profiles represented in top, middle and bottom rows formed the basis for the semi-quantitative classification of proteins presented in Table 1 and 2: not transferred (−, fold induction around <1.5), poorly transferred (+, fold induction in the range (1.5–3) and efficiently transferred (++, fold induction >3). Note that, in most cases, preferential transfer of proteins was analyzed in parallel with CD4^+^, CD8^+^ and B cells allowing comparison not only between the various proteins but also between the effector cells themselves. Similar results were obtained in 2 other experiments.

We then looked if we could detect differences in the capture of the fluorescent proteins by T or B cells during trogocytosis. As seen in Figure 3A and Table 1, no significant transfer was detectable for any of the proteins that reside in intracellular compartments such as the nucleus, cytosol, or intracellular organites. Conversely, all the proteins predicted to localize at the PM were transferred, albeit with variable efficiencies: whilst the transfer of some was easily detectable, for others only a moderate transfer was observed (Figure 3A and Table 1). Very comparable results were obtained when trogocytosis by OT-II T helper cells (Figure 3B and Table 1) or when MD4 B cells (Figure 3C and Table 1) were performed. Note that in the case of GFP-H-Ras, some non-specific transfer (i.e. transfer in the absence of triggering mAb) was noticed towards all the effector cells, reminiscent of a recent report describing the spontaneous passage of H-Ras to fresh human T lymphocytes [34]. This transfer was nevertheless greatly increased in the presence of mAb (see Figure 3 and Table 1). We found a similar capacity of CD4-GFP to transfer spontaneously to B cells (Figure 3C), but, quite remarkably, not to T cells (Figure 3A and B). When trogocytosis by T cells was triggered by the Y3 mAb, however, CD4 was then transferred very efficiently (Figure 3A and B and Table 1). The reason for this difference in CD4-GFP capture between B and T cells remains to be elucidated. Note that in all cases, the capture of FcγRII was determined and was not detectably affected by the nature of the GFP-tagged molecule expressed (see Figure 1 and data not shown). Altogether, our results reveal that quantitative differences do exist in the transfer efficiency of individual proteins from target cells to T or B cells, with a remarkable degree of similarity in the efficiency of transfer of all the various proteins to the three types of lymphocytes.

### A similar pattern of protein transfer occurs upon antigen-triggered trogocytosis

**Figure 4:**
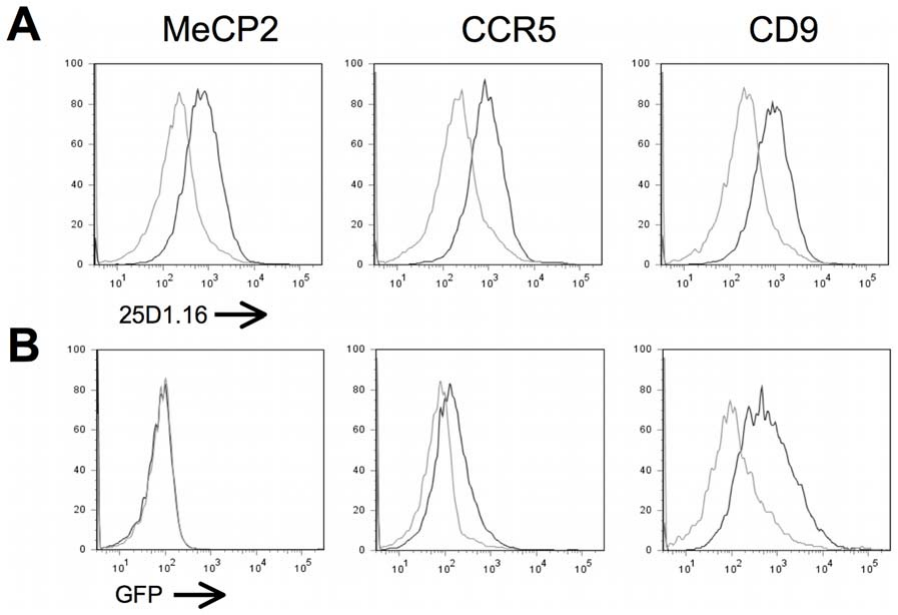
Quantitative differences in the transfer efficiency of various GFP proteins on T or B cells during Ag-mediated trogocytosis. A) OT-I T cells were co cultured with HEK-H-2K^b^-β2m-OVA cells that were transiently transfected 48 hours earlier with vectors encoding the indicated protein fused to GFP. The histograms show the capture by OT-I cells of the peptide-MHC complex (recognised by the 25D1.16 mAb), during the co culture with the target cells in the presence (white line) or absence (grey line) of latrunculin B. Left, middle and right panels provide typical examples of the results obtained respectively for proteins not transferred, poorly transferred or efficiently transferred onto OT-I T cells. B) As in A) except that GFP fluorescence was analyzed on gated OT-I T cells. Similar results were obtained for all the constructs shown on Table 1 and 2, and reproduced in 2 independent experiments.

Next, we evaluated if preferential transfer also occurred in trogocytosis triggered by antigen recognition. For that, a clone of HEK cells stably expressing the OVA antigen covalently linked to H-2K^b^ molecules and to β_2_m was transiently co-transfected with the set of constructs presented in Table 1. These HEK transfectants were then exposed to OT-I T cells, in the absence or presence of latrunculin B, which is known to block trogocytosis in T cells via inhibition of conjugate formation between T cells and their cellular partners [35]. Capture of the H-2K^b^-β_2_m-OVA complex by OT-I T cells was detected by the 25D1.16 mAb and provided a similar internal control of trogocytosis efficiency as for FcγRII capture in Figure 1. As shown in Figure 4 for three representative examples, we found that capture of the H-2K^b^-β_2_m-OVA complex was comparable whatever construct transfected in HEK cells and was blocked in the presence of latrunculin B, proving that the overall Ag-mediated trogocytosis was not affected by overexpressed proteins (Figure 4A, and data not shown). For the GFP-tagged proteins tested, the pattern of capture by OT-I T cells was very comparable to what was found in the case of redirected trogocytosis, as shown for representative examples of proteins transferred either efficiently (CD9), or moderately (CXCR5) or not at all (MeCP2) (Figure 4B, and data not shown).

### The transfer efficiency of a given protein by trogocytosis is neither directly related to its level of expression by target cells, nor to the proportion of the protein present at the PM

Although the results described above do show quantitative differences in the efficiency of transfer of various PM proteins, it remained unclear if these differences truly represented selectivity, since two other factors could potentially influence the transfer efficiency of a given PM protein: i) its level of expression on the target, and ii) its distribution between the PM and other intra-cellular compartments.

**Figure 5:**
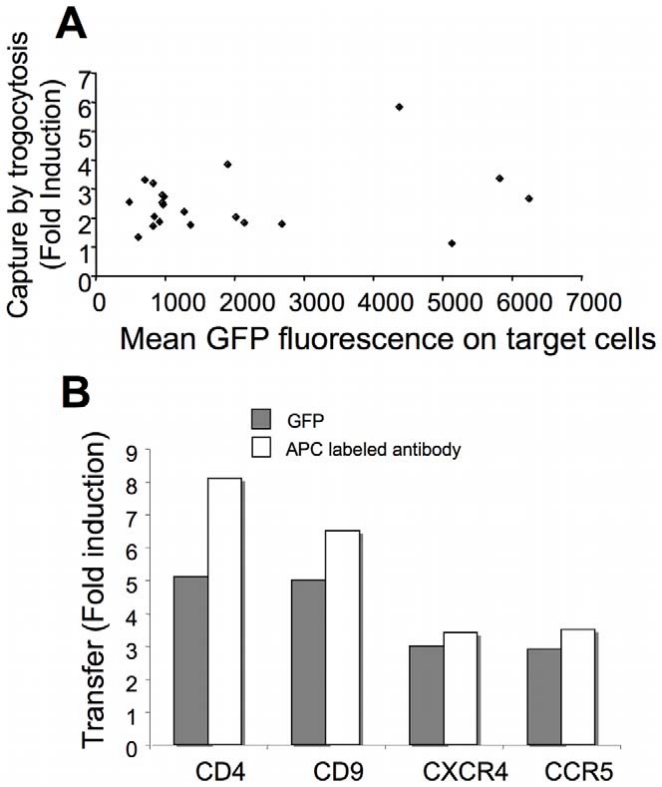
The transfer efficiency of a given protein by trogocytosis is not directly related to its level of expression by target cells nor to its proportion present at the PM. A) Mean fluorescence intensities of the expression of the indicated proteins fused to GFP by targets is plotted as function of the transfer efficiency (fold induction) on OT-I cells. Both values were obtained from cells originating from the same transfection well. B) The transfer efficiency (fold induction) on OT-I T cells of the indicated proteins expressed in HEK-FcγRII as GFP-fusions was analyzed using either GFP fluorescence (grey histograms) or mAb specific for each molecule (white histograms). Similar results were obtained in a second experiment.

To address the impact of the expression level of proteins on their transfer efficiency, we proceeded via several ways. Firstly, as shown in Figure 5A and as anticipated from the results presented in Figure 2 and Figure S1, we found that there was no global correlation between the level of expression of a protein and its transfer efficiency onto OT-I T cells: some poorly expressed proteins are efficiently transferred while other ones, efficiently expressed, are transferred very poorly. Similar results were obtained on OT-II CD4 T cells and MD4 B cells (not shown). We also analyzed the transfer efficiency of various proteins for which we manipulated the level of expression either by sorting clones expressing different levels or by using different transfection conditions (Figure S2 and Comment S1). The conclusion from these experiments is that above a certain level of expression, no increase in transfer efficiency could be evidenced.

To analyze the influence of distribution of the protein between the PM and the intra-cellular compartments, we also assessed the capture of proteins for which we had specific mAb directed against extracellular epitopes at our disposal. In this case, only the fraction of PM proteins present at the membrane, not total protein, is analyzed. Using mAb instead of the overall GFP fluorescence to detect capture, we confirmed that CD4 and CD9 transferred more efficiently than CXCR4 or CCR5 (Figure 5B). We also used a morphologic approach to quantify the ratio between the expression at the PM and in intracellular compartments for various proteins and found that this ratio was not higher in the case of proteins efficiently transferred (Figure S3 and Comment S2).

Previous papers have demonstrated a critical role for actin cytoskeleton during trogocytosis [32], [35], [36], and ectopically expressed proteins could potentially have influenced transfer efficiency through alterations of the cytoskeleton of transfected cells. Our results in Figure 1E did, however, indicate that all transfected HEK cells behaved similarly in trogocytosis assays, whatever AFP-protein was expressed. We did not, therefore, consider that it was worthwhile to explore this possibility by other means than by showing that latrunculin abolished trogocytosis (Figure 4 and not shown).

### Impact of the manipulation of anchoring motifs present in proteins on their preferential transfer

As evidenced in Figure 3 and Table 1, all PM proteins were transferred onto T and B cells during trogocytosis although with very different efficiencies. A higher efficiency for proteins anchored in the internal leaflet of the PM compared to proteins of external leaflet of the PM was frequently observed. A protein recruited to the internal leaflet of the PM via interaction with phospholipids (PH domain of PLC-δ1 fused to GFP) was also found to transfer efficiently, whereas no transfer was detected for the PH domains of other proteins that transiently interact either with the nuclear membrane (CFP-Grp) or with endosomes (GFP-PX). Finally, among transmembrane proteins, all tested members of the tetraspanin family were found to transfer efficiently, as well as CD4 and FcRγ, but others transferred only modestly (including proteins with seven transmembrane segments).

These results suggest that the mode of anchoring of a given protein to the PM is likely to play a major role in determining its transfer efficiency. If this is true, the anchoring motif of a protein should be sufficient to recapitulate the characteristics of GFP transfer onto T and B cells during trogocytosis. To test this prediction we studied GFP proteins targeted to the PM using minimal anchor motifs corresponding to those found in proteins efficiently or inefficiently transferred. As shown in Table 2, we observed that the transfer efficiency of GFP-CD59 (which is anchored to the PM via a glycophosphatidyl moiety) was comparable to that of GFP fused only to a GPI-anchor motif (GFP-GPI). Similarly, GFP carrying just the CAAX box of H-Ras was as efficiently transferred as GFP-H-ras (Table 2). Regarding members of the src-kinase family, we found that Fyn-GFP or GFP modified just by the fyn anchor were only modestly transferred during trogocytosis. Interestingly, all the members of this family were modestly transferred, although their anchors differed in the presence of charged residue (Fyn: neutral; Yes: two positive charges; Lck: two negative charges; GAP-43: three positive charges) [37]. Furthermore the fusion of GFP to a mutated form of fyn anchor (FynG2A), which was located in the cytosol rather than at the PM, did not transfer during trogocytosis (Table 2). These examples therefore show that the moiety responsible for the PM anchoring of a protein seems necessary and sufficient to allow (and possibly to predict) the transfer efficiency of PM proteins during trogocytosis.

### Capture of the efficiently transferred FcRγ-GFP protein is a suitable marker to identify antigen-specific CTL by TRAP assays

**Figure 6:**
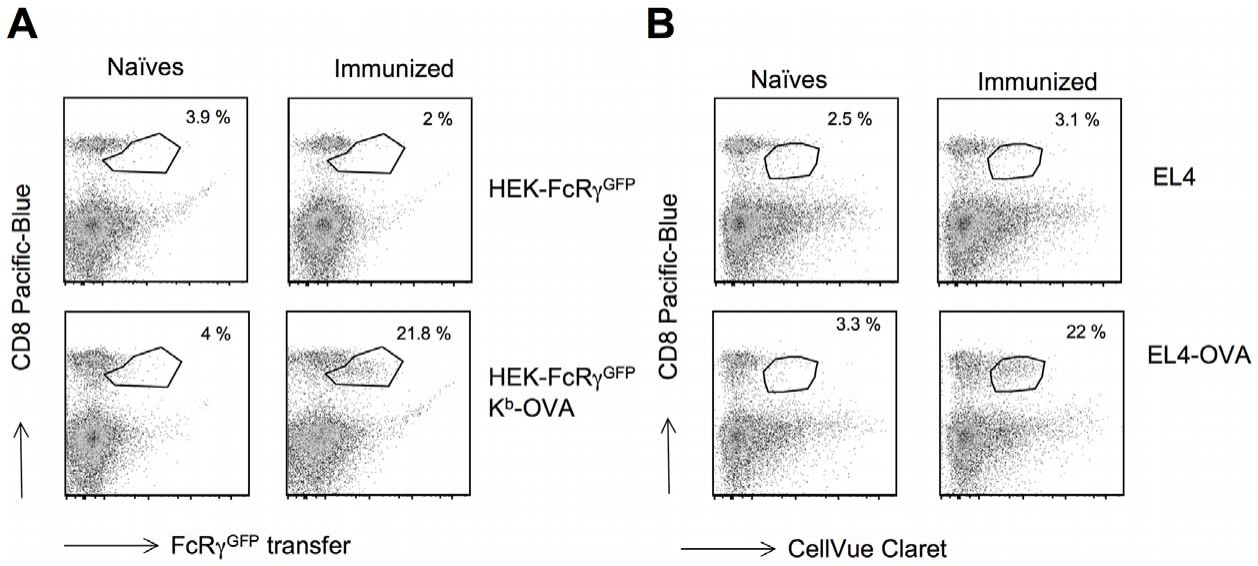
An efficiently transferred protein can be used in immunomonitoring assays to detect reactive T cells in TRAP assays. A) Splenocytes from B6 animals immunized (right panels) or not (left panels) with CyaA-OVA were co cultured with the B8 HEK-FcRγ-GFP cells (stably expressing high levels of FcRγ-GFP) transiently transfected 48 hours earlier with a vector encoding covalent H-2K^b^-β2m-OVA (bottom panels) or left untransfected (top panels). At the end of the co-culture, cells were analyzed by flow cytometry and GFP fluorescence is plotted as a function of CD8 staining. Numbers represent the percentage of CD8^+^ cells expressing GFP (present in the gate drawn in the graph). B) As in A except that splenocytes were incubated with EL4 cells labelled with the fluorescent lipophilic probe CellVue Claret loaded (bottom panels) or not (top panels) with the OVA peptide. The Fluorescence signal from CellVue Claret was analyzed as a function of CD8 staining. Numbers represent the percentage of CD8^+^ cells expressing CellVue Claret fluorescence (falling within the gate drawn in the graph).

In previous studies, we found it possible to identify reactive CTL via the capture of lipophilic probes incorporated in the PM of APC expressing their cognate antigen [7], [9], [28]. In the context of the findings reported here, we determined if the capture of one of the more efficiently captured molecule could be used in TRAP assays instead of lipophilic probes. For this, we used CTL responding to an immunization with an adenylate-cyclase (CyaA) vector carrying the immunodominant H-2K^b^-restricted OVA 257-264 peptide of ovalbumin [7], [9], [28]. Total splenocytes from immunized or control naïve B6 mice were incubated with HEK cells stably expressing FcRγ-GFP, which had been transiently transfected or not with a construct encoding H-2K^b^-OVA-β2m construct [38]. As a control, we also used EL4 cells labelled with a fluorescent lipophilic probe and pulsed with the OVA peptide as target cells. As shown in Figure 6A, we indeed found that we could identify around 20% of CD8+ T cells displaying FcRγ-GFP fluorescence after exposure of splenocytes from immunized mice to HEK-FcRγ-GFP cells expressing the H-2K^b^-OVA-β_2_m antigen. Interestingly, this proportion of OVA reactive CTL was quite comparable to that of CTL displaying DiO staining after exposure to DiO-labelled EL4 cells pulsed with the OVA antigen (Figure 6B) or to CTL stained with the H-2K^b^-OVA tetramer (not shown) or producing IFN-γ upon antigenic stimulation (not shown). Thus our results show that TRAP assays based on the capture of preferentially transferred GFP-tagged proteins permit the identification of reactive CTL within a complex mixture of effector cells with similar efficiencies to other previously documented methods.

## Discussion

In this study, based on the measurements of the transfer efficiency by trogocytosis of a series of fluorescent proteins, we found that only proteins present at the PM were detectably transferred, and evidenced some degree of selectivity in the transfer of PM-associated proteins during trogocytosis. We believe our findings could not only help to distinguish between the potential mechanisms proposed for trogocytosis but also be exploited for the design of novel immunomonitoring tools.

Our results that proteins not associated to the PM are not transferred during trogocytosis (Figure 3 and Table 1) are compatible with previous results obtained with cytosolic dyes such as calcein, CMTMR or CFSE, which were all shown to transfer very poorly during trogocytosis performed by T or NK cells [7], [8]. Here we have shown that no transfer could be detected for nuclear proteins such as MeCP2 but also for proteins present predominantly in intracellular organelles such as the endoplasmic reticulum, lysosomes or endosomes (p61HcK) or the cytosol (actin or myosin). In contrast, proteins present at the PM were all transferred, although with different efficiencies. Those included integral membrane proteins, as well as others anchored to the internal or external PM leaflet, but also the PH domain of PLC-δ1, which is not directly anchored to the PM, but transiently recruited to its cytoplasmic face through interaction with membrane phospholipids [39].

Among those proteins present at the PM of target cells, however, we found major differences in the transfer efficiency (Table 1). Disappointingly, but also interestingly, no obvious rule emerged to explain why a given PM protein should transfer efficiently or not. We are aware that the approach we chose is only relative, and does not allow an absolute quantification of the transfer efficiency. Thus, we could measure if the transfer of a given protein, overexpressed in target cells (Figure S1), was easy or hard to detect or could not be detected at all. As each different protein could modify target cell recognition by T cells, we first ensured that trogocytosis, as measured independently by the capture of FcγRII, was not being affected, whatever AFP-tagged protein was being expressed by the target cells (Figure 1). We are thus confident that the differences in transfer efficiency we observed were not the result of an altered recognition of the target cells. Second, for a set of selected proteins, we found that above a certain threshold, the efficiency of trogocytosis reaches a plateau, and a further increase of the expression level of the fluorescent protein by target cells does not result in an increase in the amounts of protein being transferred (Figure 5, Figures S2 and S3 and Comments S1 and S2). So, we are confident that this parameter only moderately impacts our conclusions since we only chose proteins that were expressed at very high levels. Finally, for the proteins that are associated to the PM, we found no clear-cut correlation of the transfer efficiency with the proportion of the protein associated to the PM (Figure S3 and Comment S2). We are therefore confident that the differences in transfer efficiency we have found are the reflection of preferential transfer, and we have tried to understand what molecular basis this selectivity could have. Furthermore, studies from other laboratories have previously documented the transfer of several proteins expressed naturally, and those were all among the set of proteins which we found to be preferentially transferred to T or B cells from transiently transfected HEK cells (see the cases of Ras, CD4, CD9 described below). The fact that these proteins were shown to transfer in conditions where they were not over-expressed is an indirect confirmation that the higher transfer efficiency is the reflection of preferential transfer.

With regard to the involvement of cholesterol-rich membrane microdomains, we observed that proteins thought to be included (HA or H-Ras for instance) or excluded from these domains (VSVG or K-Ras for instance) (see [40] and references therein) could be either efficiently (H- and K-Ras) or inefficiently (HA and VSVG) transferred. Thus, the tendency of a protein to associate to rafts or to be excluded from them does not provide an explanation to our observations. Note that cholesterol-depleting reagents such as methyl-β-cyclodextrin have no effect on trogocytosis [[41] and our unpublished observations] thus reinforcing the notion that, globally, rafts are not essential for this process. Unexpectedly, we found that proteins attached to the PM external leaflet, such as GPI-anchored proteins (GFP-CD59, GFP-GPI), were inefficiently transferred, whereas proteins anchored to the internal leaflet were often transferred more efficiently (H-ras, K-ras, p59HcK, kinases of the src-family in general), as well as PH-PLC-δ1, which only associates to the internal leaflet transiently via phosphoinositides (Figure 3 and Table 1). These observations are quite counter-intuitive since proteins in the internal leaflet are not in direct contact with the interacting lymphocyte and GPI-anchored proteins have been shown to be frequently transferred to adjacent cells via a mechanism called GPI-painting (see [42] and references therein). It could well be, however, that different mechanisms are involved for trogocytosis and GPI-painting. Regarding the efficient transfer of proteins anchored to the PM internal leaflet, we are still looking for a potential explanation, but it is possible that, during the time of target cell-effector cell interaction, proteins present in the internal leaflet exhibit a higher mobility than their external leaflet counter-parts [a concept supported by the work of Kenworthy et al. [40]], which would likely be engaged in protein-protein interactions with the surface molecules of lymphocytes. Thus, preferential capture of proteins attached to the internal leaflet of the PM may reflect higher dynamics of this compartment.

Noticeably, we found that all the members of the tetraspanin family we tested (CD9, CD81 and CD82) were amongst the most efficiently transferred proteins (Figure 3 and Table 1). Interestingly, members of this family have already been shown to transfer efficiently between cells in the course of the interaction between dendritic cells and T cells [43] and between ovocyte and sperm [44], [45], via mechanisms related to trogocytosis. This could illustrate a particular role played by the tetraspanin-microdomains in trogocytosis. Those domains are known as «organizers» of the PM in that they concentrate particular proteins and lipids through direct or indirect association to tetraspanins [46], a phenomenon particularly well described for leukocytes [47]. The precise composition of these domains, however, still remains to be elucidated because the molecular approaches to isolate them are at least partly overlapping with those for the isolation of cholesterol-rich membrane microdomains. Interestingly, apart from tetraspanins, two of the proteins we found to be most efficiently transferred, H-Ras and CD4, were both previously reported to be captured by T cells from target cells expressing them naturally [20], [34], [48], which constitutes an argument supporting the fact that these proteins are, indeed, more efficiently transferred than others, and against the possibility that the conditions of overexpression used in our study may have induced artefacts. Note that the fact that proteins present in the internal leaflet as well as integral membrane proteins such as tetraspanins, thought to have no ligand, are preferentially transferred during trogocytosis strongly suggests that the preferential transfer of a protein is not due to a direct interaction with a counter-ligand on the lymphocyte surface.

Interestingly we found that, in several cases, the anchor domain of the proteins was a critical determinant of the transfer efficiency (Table 2). A particularly striking example was when the anchor domain of Fyn was mutated such that it did not allow lipidic modifications of Fyn; the resulting protein was confined to the cytosol and did not transfer by trogocytosis any more. This type of situation is also well illustrated by the two isoforms of HcK, another member of the src-kinase family. In the wild type protein, p59HcK and p61HcK solely differ by the 21 N-terminal amino acids due to alternative initiation of translation resulting in a modification of the lipid modifications and of their localization, p61 being in the lysosomes and p59 at the PM [49]. In our experiments, we found that p61-GFP does not transfer by trogocytosis whereas p59-GFP does. When GFP was fused just to the anchor motifs of src kinases, such as those of Yes or Lck, this also led to proteins being transferred by trogocytosis. In the case of Ras, we found that the CAAX box of H-Ras or K-Ras fused to GFP recapitulated the transfer efficiency of GFP-H-Ras- or GFP-K-Ras. Finally we found that both GFP-CD59, which is GPI-anchored, and a GPI-anchored GFP were poorly transferred suggesting that GPI-anchored proteins are not enriched in the membrane fragments captured by trogocytosis. All these results suggest that PM localization is the key element determining the actual occurrence of transfer of a protein during trogocytosis, and that the nature of the PM anchor can determine a certain degree of selectivity during this transfer.

Note that the proteins analyzed in this study were not chosen for their particular relevance in cellular interactions involving T and/or B cells. Rather, most of them had been extensively characterized for the role of their anchor motif in determining their subcellular location and were therefore very appropriate probes for our purpose of linking transfer efficiency and subcellular location. Given the technical difficulties of transfecting primary T and B cell lines, a possible way to investigate protein transfer in natural interactions involving T and/or B cells rests on the use of mAb to known molecules. Results obtained along this line confirmed that several of the proteins identified in our study (or belonging to the same family of protein) indeed exchange efficiently during trogocytosis ([44], [45] and see also [50] for a review). However, these studies, based on the use of mAb recognizing extracellular epitopes could not reveal the preferential transfer of proteins of the internal PM leaflet. Yet, in one of the first studies documenting trogocytosis, the transfer of membrane fragment was revealed by the exchange of YFP modified by the palmitoylation motif of neuromodulin (GAP-43), which targets it to the internal leaflet of the PM [14].

Trogocytosis can be triggered by interactions involving multiple receptors (see [6], [51] as illustrations). In our case, we analyzed trogocytosis triggered by an identified stimulus such as mAb or antigen on T or B cells. However, our observation that CD4 is spontaneously captured by B cells in the absence of stimulation (Figure 3C) likely reflects the involvement of additional receptors (expressed on B but not T cells), the identity of which remains to be elucidated (with MHC class II molecules being possible candidates).

How can we use these findings to better understand the mechanisms of trogocytosis? Several models have been proposed to explain the transfer of proteins by trogocytosis such as those involving proteolytic cleavage, up-rooting, membrane bridges, nanotubes, exosomes/microvesicles or detachment of PM fragments or villi [1], [23]. The transfer of full-length proteins or of proteins present only in the internal leaflet of the membrane clearly rule out proteolytic cleavage and up rooting as possible mechanisms for trogocytosis. Regarding membrane bridges and nanotubes, what kind of components can be transferred via such structure still remains unclear, and this is further complicated by the fact that various types of nanotubular structures have been described [52]. Some of those were shown to allow for the transfer of cytosolic proteins [52], implying the existence of a continuity between the cytoplasms of the donor and the recipient cell, which would not comply with our observations that cytoplasmic components do not get transferred via trogocytosis. In contrast, other types of nanotubes did not allow for the transfer of cytosolic components and in fact, for those latter types of nanotubes, no continuity between the membrane of the two connected cells could be established [52]. Thus, from what is currently known about transfer mediated by nanotubes, this last type of structure could be compatible with the results obtained in our study.

Exosomes are another potential vector of trogocytosis. Thanks to biochemical and proteomic studies, the protein composition of exosomes and microvesicles is relatively well known [25], [53]. Interestingly MHC molecules (known to transfer during trogocytosis [13]) but also tetraspanins are among the quantitatively major proteins found in these structures [25], [43], [54]. However, these structures also appear to be rich in actin and myosin [25], [53], [54], which, we found, did not transfer to effector cells during trogocytosis. Furthermore, one study reported on the absence of FcR from exosomes/microvesicles obtained from dendritic cells [54], and we found FcγRII/III and FcRγ-GFP to be efficiently transferred via trogocytosis [Table 1 and [33]]. Although dedicated studies clearly need to be performed to fully address the issues of the molecular structures involved in the transfer of PM-associated materials during trogocytosis, our results provide arguments against various potential pathways such as exosomes or nanotubes leading to the establishment of cytoplasmic continuity. By elimination, our results therefore bring support in favour of either detachment of PM fragments, possibly in a vesicular form [55], and/or closed ended nanotubes as the mechanism of trogocytosis. In line with this last hypothesis, a recent study by the group of Davis showed that microvilli could be fragile areas that are preferentially captured by NK cells [56]. Similar conclusions could be drawn from the study by Pardigon et al. which showed snatching of the TL molecule by CD8αα T cells [6]. Indeed, this study showed that patches of acquired TL molecules were present on lymphocytes after capture in “plates” similar in dimensions to what is observed at the point of contact between lymphocytes and target cells. The fact that those “plates” remained as organized membrane domains following transfer indicates that diffusion of transferred TL is highly limited. These results also make it unlikely that the TL molecule could be transferred via secreted vesicular structures (e.g., exosomes) or tunnelling nanotubes and are therefore in line with our own results.

A major difficulty to distinguish between all these mechanisms is the lack of molecular tools to selectively block the formation of nanotubes, exosomes or microvilli. Such is also the case for trogocytosis. In the absence of such tools, we believe that our study opens new perspectives on the identification of the mechanism of trogocytosis by providing a set of testable predictions based on the panel of transferred versus non-transferred molecular probes.

A key question about trogocytosis concerns the role played by captured molecule on recipient T or B cells (see [1], [2] for reviews on such proposed roles). Our study provides a series of proteins including ligands, receptors, signalling proteins or membrane organizing proteins which can be tested for their biological function on recipient T or B cells. Whether functions harboured by these proteins on donor cells will be fully or partially recapitulated in recipient cells is a very important question to address both for mechanistic and functional reasons. Indeed, it is still unclear if captured molecules insert properly in the plasma membrane of recipient cells, which will directly condition the functions they can fulfil [see [33] and references therein for instance].

From a technological point of view, TRAP assays have proven useful to detect, characterize and purify antigen-reactive lymphocytes [28]. Indeed, antigen-reactive lymphocytes can be distinguished from the non-reactive ones by their propensity to capture PM components from target cells expressing the antigen. These assays were used to identify, characterize and purify lymphocytes reactive against viral infection [27], tumor cells [29] and vaccine [7], [9], [28] both in the mouse and human systems. So far, these assays were mostly performed using target cells that were loaded with lipophilic dyes or that were surface biotinylated [7], [9], [27]–[29], [57]. We show here that similar results can be obtained with target cells expressing preferentially transferred fluorescent proteins such as FcRγ-GFP (Figure 6). Although, in the context of this study, we used target cells transfected with plasmid constructs encoding these proteins, this type of approach would not be convenient for the wide development of TRAP assays. A more versatile way would, however, consist in using viral vectors to express a GFP construct that is efficiently transferred during trogocytosis, or by directly loading the recombinant protein into target cells using protein transduction approaches [58]. Furthermore, our finding opens the way to the construction of a mouse model for the assessment of trogocytosis *in vivo* by generating a transgenic mouse expressing a selectively transferred fluorescent protein under control of an appropriate promoter driving high levels of expression on professional APCs. Since trogocytosis is performed by many cells of our immune system, this could be a useful tool to address the important issues regarding the role of antigen exchange between APCs mediated by trogocytosis, or for the parallel identification of cells of different immune lineages that react specifically with antigen-bearing cells in the course of a given infection, vaccination or disease model.

## Materials and Methods

### Ethics statement

Mice were handled in strict accordance with good animal practice as defined by the European and French guidelines, and all animal work was approved by the ethic committee of Midi-Pyrénées (# 20080307/7).

### Mice and cell lines

Effector cells originated from OT-I mice (CD8^+^ T cells specific for OVA257–264 presented by H-2K^b^), OT-II mice (CD4^+^ T cells specific for OVA323–339 presented by I-A^d^), or MD4 mice (B cells specific for hen egg lysozyme [HEL]). To obtain T cells, total splenocytes were stimulated with the appropriate antigenic peptide (0.1 µM OVA257–264 for OT-I CD8^+^ cells and 1 µM OVA323–339 for OT-II CD4^+^ cells) and used between days 4 to 6 following stimulation. B cells from MD4 spleen were used either immediately or after an overnight culture. Activated T cells or naive B cells were exposed to HEK cells and to stable transfectants of these cells expressing either FcγRII (for redirected trogocytosis) or H-2K^b^-OVA (for antigen specific trogocytosis) [33]. Other HEK stable transfectants expressing FcRγ-GFP at different levels were sorted by flow cytometry from a cell line generated previously [33]. In some assays, the EL4 thymoma cells (H-2^b^ haplotype) were used as targets cells. All cell lines were cultured in RPMI 1640 with 10% heat-inactivated FBS, penicillin-streptomycin (100 U/ml) and 2 mM glutamine.

### Reagents, antibodies and molecular biology

Peptides were synthesized in our laboratory, HPLC-purified (>98%) and their identity confirmed by mass spectrometry. The biotinylated anti-Flag antibodies were from Sigma-Aldrich (Saint-Quentin-Fallavier, France). Fluorescently labelled mAb against mouse CD8α (53.6.7.2), CD4 (GK1.5, RM4-4 or RM4-5), B220 (R4-6B-2), FcγRII/FcγRIII (2.4G2), unlabelled mAb against murine BCR κ chain, rat IgG1 and mouse IgG2a and fluorescent streptavidin were from Becton-Dickinson/Pharmingen (Le-Pont-de-Claix, France). Unlabelled mAb to H-2K^b^ (Y3) and mAb to HLA class I (W6/32) were obtained from culture supernatant of the corresponding hybridoma. Antibodies to green fluorescent protein (GFP) were from Abcam (Paris, France).

### Construction of plasmids

Constructs encoding MeCP2 fused to GFP were obtained by subcloning the PCR-amplified cDNA of MeCP2-B [59] directly in pEGFP-N3, between the BglII and ApaI restriction sites. Construction of a vector encoding the FcRγ chain fused to GFP (FcRγ-GFP) was described previously [33]. The vector encoding murine flagged FcγRII has been described previously [31]. The complete list of the plasmids used in the study is summarized in Table 1 and 2. Cells were used in trogocytosis experiment 48 hours after transient transfections.

### Transient transfections

For transient transfection experiments, HEK cells (or sometimes stable HEK transfectants expressing FcγRII, FcRγ-GFP or H-2K^b^-OVA) were plated to reach 50% confluence in 6 well plates and transiently transfected with 2 µg of DNA and JetPEI (Ozyme) (6 µl/well), following the manufacturer recommendations. The level of expression of the given molecule was assessed by flow cytometry using a LSRII cytometer (Becton Dickinson, Mountain View, CA), and we ensured that the percentages of transfected cells were always superior to 40%.

### Trogocytosis experiments

For redirected trogocytosis, experiments were performed as described previously [32], but using HEK cells stably transfected for the expression of FcγRII instead of P815 cells. In brief, HEK-FcγRII cells, transiently transfected with the various AFP-tagged constructs, were placed in U-bottomed 96-well plates (0.5×10^6^ cells/well in 100 µl final volume). Effector T or B cells were pre-incubated or not with unlabelled antibodies triggering trogocytosis (or in some experiments, their isotype controls) [32] (Y3 anti-H-2K^b^ for OT-I and OT-II cells and anti-κ chain mAb for B cells) and conjugates were formed with HEK-FcγRII cells (0.1×10^6^ cells/well in 100 µl final volume) by centrifugation for 30 seconds at 160*g*, and then left at 37°C for 1 hour. Conjugates were then dissociated by washing cells twice in cold phosphate-buffered saline containing 0.5 mM ethylenediaminetetraacetic acid (EDTA) and pipetting them up and down thoroughly, before staining on ice with mAbs against CD8, CD4, or B220. Cells were then analyzed on a LSRII flow cytometer (Becton Dickinson). Effector cells were gated positively according to their staining with lineage-specific markers (CD8 for CTL, CD4 for T helper cells, and B220 for B cells). Transfer of GFP proteins was studied by directly following the GFP signal on effector cells. Transfer of Flag-FcγRII was followed on T cells by using 2.4.G2 antibody (against both FcγRII/III), and on B cells by using a biotinylated anti-Flag antibody followed by fluorescent streptavidin

### Calculations of trogocytosis efficiency

Fold induction (FI) of trogocytosis was calculated as the ratio of the median fluorescence intensity of GFP on gated CD4^+^, CD8^+^ T or B220^+^ B cells, measured in the presence or absence of the mAb triggering redirected trogocytosis. Values used for the measurements were median fluorescence intensities (mfi). In some cases FI of the capture of the indicated GFP proteins was normalized (normalized FI, nFI) by that of the FcγRII itself.

### Fluorescence microscopy experiments

For quantification of the PM vs. intracellular proportions of a transiently expressed GFP-tagged protein, a few transiently transfected cells were taken from those used in trogocytosis experiments, placed to adhere on coverslips and analyzed by confocal fluorescent microscopy (Leica TCS-SP2) and the Metamorph software. A mask corresponding to the PM was created using staining with anti-MHC class I mAb. This mask was then used to quantify GFP signals overlapping with the mask (plasma membrane) and those excluded from the mask (intracellular signals).

### Immunization of mice

Mice (3 per experimental condition) received two intradermal injections (seven days apart) of 20 µg of the CyaA-OVA [60]. For vaccine preparation, recombinant CyaA carrying the OVA antigen was mixed with CpG just before the injections, to obtain a solution with a final concentration of 500 µg/ml CyaA and 100 µg/ml CpG oligonucleotides (CpG 1826: TCCATGACGTTCCTGACGTT, Sigma Aldrich, Saint-Quentin Fallavier, France). Seven days after the second injection, mice were sacrificed and their splenocytes were collected and used in trogocytosis experiments.

## Supporting Information

Comment S1 (0.05 MB DOC)

Comment S2 (0.03 MB DOC)

Figure S1 Examples of the levels of expression attained after transient transfection with plasmids coding for various proteins fused to GFP. Typical examples of flow cytometry analyses 48 hours after transient transfection in HEK-FcγRII of 9 different proteins fused to GFP. This type of analysis was performed systematically for all proteins used in this study to ensure that strong expression of the GFP-tagged proteins was detected in at least 40% of HEK-FcγRII.
(0.81 MB TIF)

Figure S2 The transfer efficiency of a given protein by trogocytosis is not directly related to its level of expression by target cells. A) Expression by HEK-FcγRII cell of the FcRγ-GFP protein expressed after transient transfection with increasing amounts of vector encoding FcRγ-GFP is shown in the top panels. Capture of the FcRγ-GFP by gated OT-I cells exposed to the target cells shown in top panels in the presence (white histograms) or absence (grey histograms) of the Y3 mAb. B) The graph shows the fold induction of FcRγ-GFP capture by OT-I cells as a function of FcRγ-GFP expression on target cells, compiled from eight separate transfections attaining different levels of FcRγ-GFP expression. C) As in B) except that various amounts of vector DNA coding for CD9 (empty diamonds), CXCR4 (empty squares) or CCR5 (full circles) were used to transfect HEK-FcγRII cells.
(1.02 MB TIF)

Figure S3 Differences in the transfer efficiency of various GFP proteins on T or B cells are not correlated to their expression levels at the PM. A) A mask (middle panel) delimitating the PM of HEK-FcRγGFP was constructed using the Metamorph software based on extra-cellular anti-MHC class I staining (left panel) and was applied on FcRγ-GFP staining in order to discriminate between GFP fluorescence present at the membrane and intracellularly. B) Arbitrary fluorescent units given by the software on FcR γ-GFP present at the PM (squares) or intracellularly (diamonds) are given for 12 different slices of transfected cells (0.5µm difference between each slice). C) The ratio between cell surface versus intracellular GFP fluorescence is shown for 4 different clones of HEK-expressing increasing levels of FcRγ-GFP (numbers below the clone names refer to the ratio of the mean fluorescence intensity of total FcRγ-GFP expression for each clone divided by that of untransfected HEK cells, measured by flow cytometry). D) The ratio between cell surface versus intracellular GFP fluorescence was similarly calculated on HEK cells transfected with four different vectors encoding either CD4, CD9, CXCR5 or CCR5 fused to GFP. CD4 and CD9 were chosen as examples of proteins efficiently transferred during trogocytosis and CXCR5 and CCR5 as examples of proteins poorly transferred.
(0.90 MB TIF)

## References

[1] Davis DM (2007). Intercellular transfer of cell-surface proteins is common and can affect many stages of an immune response.. Nat Rev Immunol.

[2] Joly E, Hudrisier D (2003). What is trogocytosis and what is its purpose.. Nat Immunol.

[3] Beum PV, Kennedy AD, Williams ME, Lindorfer MA, Taylor RP (2006). The shaving reaction: rituximab/CD20 complexes are removed from mantle cell lymphoma and chronic lymphocytic leukemia cells by THP-1 monocytes.. J Immunol.

[4] Harshyne L, Zimmer M, Watkins S, Barratt-Boyes S (2003). A role for class A scavenger receptor in dendritic cell nibbling from live cells.. J Immunol.

[5] Sprent J (2005). Swapping Molecules During Cell-Cell Interactions.. Sci STKE.

[6] Pardigon N, Takeda K, Saunier B, Hornung F, Gibbs J (2006). CD8-Mediated Intraepithelial Lymphocyte Snatching of Thymic Leukemia MHC Class Ib Molecules In Vitro and In Vivo.. J Immunol.

[7] Puaux A, Campanaud J, Salles A, Preville X, Timermann B (2006). A very rapid and simple assay based on trogocytosis to detect and measure specific T and B cells reactivity by flow cytometry.. Eur J Immunol.

[8] Vanherberghen B, Andersson K, Carlin LM, Nolte-'t Hoen EN, Williams GS (2004). Human and murine inhibitory natural killer cell receptors transfer from natural killer cells to target cells.. Proc Natl Acad Sci U S A.

[9] Daubeuf S, Aucher A, Sampathkumar S, Preville X, Yarema K (2007). Chemical labels metabolically installed into the glycoconjugates of the target cell surface can be used to track lymphocyte/target cell interplay via trogocytosis: comparisons with lipophilic dyes and biotin.. Immunol Invest.

[10] Hudrisier D, Riond J, Mazarguil H, Gairin JE, Joly E (2001). Cutting edge: CTLs rapidly capture membrane fragments from target cells in a TCR signaling-dependent manner.. J Immunol.

[11] Busch A, Quast T, Keller S, Kolanus W, Knolle P (2008). Transfer of T cell surface molecules to dendritic cells upon CD4+ T cell priming involves two distinct mechanisms.. J Immunol.

[12] Roda-Navarro P, Reyburn HT (2007). Intercellular protein transfer at the NK cell immune synapse: mechanisms and physiological significance.. Faseb J.

[13] Wetzel S, Parker D (2006). MHC Transfer from APC to T Cells Following Antigen Recognition.. Crit Rev Immunol.

[14] Stinchcombe JC, Bossi G, Booth S, Griffiths GM (2001). The immunological synapse of CTL contains a secretory domain and membrane bridges.. Immunity.

[15] Onfelt B, Nedvedtzki S, Yanagi K, Davis D (2004). Membrane nanotubes connect immune cells.. J Immunol.

[16] Sowinski S, Jolly C, Berninghausen O, Purbhoo MA, Chauveau A (2008). Membrane nanotubes physically connect T cells over long distances presenting a novel route for HIV-1 transmission.. Nat Cell Biol.

[17] Watkins SC, Salter RD (2005). Functional connectivity between immune cells mediated by tunneling nanotubules.. Immunity.

[18] Hwang I, Shen X, Sprent J (2003). Direct stimulation of naive T cells by membrane vesicles from antigen-presenting cells: distinct roles for CD54 and B7 molecules.. Proc Natl Acad Sci U S A.

[19] Patel D, Dudek R, Mannie M (2001). Intercellular exchange of class II MHC complexes: ultrastructural localization and functional presentation of adsorbed I-A/peptide complexes.. Cell Immunol.

[20] Patel DM, Arnold PY, White GA, Nardella JP, Mannie MD (1999). Class II MHC/peptide complexes are released from APC and are acquired by T cell responders during specific antigen recognition.. J Immunol.

[21] Patel DM, Mannie MD (2001). Intercellular exchange of class II major histocompatibility complex/peptide complexes is a conserved process that requires activation of T cells but is constitutive in other types of antigen presenting cell.. Cell Immunol.

[22] Arnold PY, Mannie MD (1999). Vesicles bearing MHC class II molecules mediate transfer of antigen from antigen-presenting cells to CD4+ T cells.. Eur J Immunol.

[23] Hudrisier D, Bongrand P (2002). Intercellular transfer of antigen-presenting cell determinants onto T cells: molecular mechanisms and biological significance.. Faseb J.

[24] Thery C, Ostrowski M, Segura E (2009). Membrane vesicles as conveyors of immune responses.. Nat Rev Immunol.

[25] Thery C, Zitvogel L, Amigorena S (2002). Exosomes: composition, biogenesis and function.. Nat Rev Immunol.

[26] Davis D, Sowinski S (2008). Membrane nanotubes: dynamic long-distance connections between animal cells.. Nat Rev Mol Cell Biol.

[27] Beadling C, Slifka M (2006). Quantifying viable virus-specific T cells without a priori knowledge of fine epitope specificity.. Nat Med.

[28] Daubeuf S, Puaux A, Joly E, Hudrisier D (2007). A simple trogocytosis-based method to detect, quantify, characterize and purify antigen-specific live lymphocytes by flow cytometry, via their capture of membrane fragments from antigen-presenting cells.. Nat Prot.

[29] Machlenkin A, Uzana R, Frankenburg S, Eisenberg G, Eisenbach L (2008). Capture of tumor cell membranes by trogocytosis facilitates detection and isolation of tumor-specific functional CTLs.. Cancer Res.

[30] Tomaru U, Yamano Y, Nagai M, Maric D, Kaumaya PT (2003). Detection of virus-specific T cells and CD8(+) T-cell epitopes by acquisition of peptide-HLA-GFP complexes: analysis of T-cell phenotype and function in chronic viral infections.. Nat Med.

[31] Mancardi DA, Iannascoli B, Hoos S, England P, Daeron M (2008). FcgammaRIV is a mouse IgE receptor that resembles macrophage FcepsilonRI in humans and promotes IgE-induced lung inflammation.. J Clin Invest.

[32] Hudrisier D, Aucher A, Puaux AL, Bordier C, Joly E (2007). Capture of Target Cell Membrane Components via Trogocytosis Is Triggered by a Selected Set of Surface Molecules on T or B Cells.. J Immunol.

[33] Hudrisier D, Clemenceau B, Balor S, Daubeuf S, Magdeleine E (2009). Ligand binding but undetected functional response of FcR after their capture by T cells via trogocytosis.. J Immunol.

[34] Rechavi O, Goldstein I, Vernitsky H, Rotblat B, Kloog Y (2007). Intercellular transfer of oncogenic h-ras at the immunological synapse.. PLoS ONE.

[35] Aucher A, Magdeleine E, Joly E, Hudrisier D (2008). Capture of plasma membrane fragments from target cells by trogocytosis requires signaling in T cells but not in B cells.. Blood.

[36] Hwang I, Sprent J (2001). Role of actin cytoskeleton in T cell absorption and internalization of ligands from APC.. J Immunol.

[37] McCabe JB, Berthiaume LG (1999). Functional roles for fatty acylated amino-terminal domains in subcellular localization.. Mol Biol Cell.

[38] Yu YY, Netuschil N, Lybarger L, Connolly JM, Hansen TH (2002). Cutting edge: single-chain trimers of MHC class I molecules form stable structures that potently stimulate antigen-specific T cells and B cells.. J Immunol.

[39] Varnai P, Balla T (1998). Visualization of phosphoinositides that bind pleckstrin homology domains: calcium- and agonist-induced dynamic changes and relationship to myo-[3H]inositol-labeled phosphoinositide pools.. J Cell Biol.

[40] Kenworthy AK, Nichols BJ, Remmert CL, Hendrix GM, Kumar M (2004). Dynamics of putative raft-associated proteins at the cell surface.. J Cell Biol.

[41] Quah BJ, Barlow VP, McPhun V, Matthaei KI, Hulett MD (2008). Bystander B cells rapidly acquire antigen receptors from activated B cells by membrane transfer.. Proc Natl Acad Sci U S A.

[42] Liu T, Li R, Pan T, Liu D, Petersen RB (2002). Intercellular transfer of the cellular prion protein.. J Biol Chem.

[43] Buschow SI, Nolte-'t Hoen EN, van Niel G, Pols MS, ten Broeke T (2009). MHC II in dendritic cells is targeted to lysosomes or T cell-induced exosomes via distinct multivesicular body pathways.. Traffic.

[44] Barraud-Lange V, Naud-Barriant N, Bomsel M, Wolf JP, Ziyyat A (2007). Transfer of oocyte membrane fragments to fertilizing spermatozoa.. Faseb J.

[45] Miyado K, Yamada G, Yamada S, Hasuwa H, Nakamura Y (2000). Requirement of CD9 on the egg plasma membrane for fertilization.. Science.

[46] Hemler ME (2003). Tetraspanin proteins mediate cellular penetration, invasion, and fusion events and define a novel type of membrane microdomain.. Annu Rev Cell Dev Biol.

[47] Tarrant JM, Robb L, van Spriel AB, Wright MD (2003). Tetraspanins: molecular organisers of the leukocyte surface.. Trends Immunol.

[48] Hudrisier D, Riond J, Garidou L, Duthoit C, Joly E (2005). T Cell activation correlates with an increased proportion of antigen among the materials acquired from target cells.. Eur J Immunol.

[49] Carreno S, Gouze ME, Schaak S, Emorine LJ, Maridonneau-Parini I (2000). Lack of palmitoylation redirects p59Hck from the plasma membrane to p61Hck-positive lysosomes.. J Biol Chem.

[50] Smyth LA, Afzali B, Tsang J, Lombardi G, Lechler RI (2007). Intercellular transfer of MHC and immunological molecules: molecular mechanisms and biological significance.. Am J Transplant.

[51] Hwang I, Huang JF, Kishimoto H, Brunmark A, Peterson PA (2000). T cells can use either T cell receptor or CD28 receptors to absorb and internalize cell surface molecules derived from antigen-presenting cells.. J Exp Med.

[52] Davis DM, Sowinski S (2008). Membrane nanotubes: dynamic long-distance connections between animal cells.. Nat Rev Mol Cell Biol.

[53] Miguet L, Pacaud K, Felden C, Hugel B, Martinez MC (2006). Proteomic analysis of malignant lymphocyte membrane microparticles using double ionization coverage optimization.. Proteomics.

[54] Thery C, Boussac M, Veron P, Ricciardi-Castagnoli P, Raposo G (2001). Proteomic analysis of dendritic cell-derived exosomes: a secreted subcellular compartment distinct from apoptotic vesicles.. J Immunol.

[55] Cocucci E, Racchetti G, Meldolesi J (2009). Shedding microvesicles: artefacts no more.. Trends Cell Biol.

[56] Williams GS, Collinson LM, Brzostek J, Eissmann P, Almeida CR (2007). Membranous structures transfer cell surface proteins across NK cell immune synapses.. Traffic.

[57] Daubeuf S, Bordier C, Hudrisier D, Joly E (2008). Suitability of various membrane lipophilic probes for the detection of trogocytosis by flow cytometry.. Cytometry.

[58] Joliot A, Prochiantz A (2004). Transduction peptides: from technology to physiology.. Nat Cell Biol.

[59] Mnatzakanian GN, Lohi H, Munteanu I, Alfred SE, Yamada T (2004). A previously unidentified MECP2 open reading frame defines a new protein isoform relevant to Rett syndrome.. Nat Genet.

[60] Fayolle C, Ladant D, Karimova G, Ullmann A, Leclerc C (1999). Therapy of murine tumors with recombinant Bordetella pertussis adenylate cyclase carrying a cytotoxic T cell epitope.. J Immunol.

[61] Joly E (2007). Optimising Blue Fluorescent Protein (BFP) for use as a mammalian reporter gene in parallel with Green Fluorescent Protein (GFP).. Nat Precedings.

[62] Hernandez LD, Hueffer K, Wenk MR, Galan JE (2004). Salmonella modulates vesicular traffic by altering phosphoinositide metabolism.. Science.

[63] Nichols BJ, Kenworthy AK, Polishchuk RS, Lodge R, Roberts TH (2001). Rapid cycling of lipid raft markers between the cell surface and Golgi complex.. J Cell Biol.

[64] Choy E, Chiu VK, Silletti J, Feoktistov M, Morimoto T (1999). Endomembrane trafficking of ras: the CAAX motif targets proteins to the ER and Golgi.. Cell.

[65] Toomre D, Keller P, White J, Olivo JC, Simons K (1999). Dual-color visualization of trans-Golgi network to plasma membrane traffic along microtubules in living cells.. J Cell Sci.

[66] Bunnell SC, Hong DI, Kardon JR, Yamazaki T, McGlade CJ (2002). T cell receptor ligation induces the formation of dynamically regulated signaling assemblies.. J Cell Biol.

[67] Pralle A, Keller P, Florin EL, Simons K, Horber JK (2000). Sphingolipid-cholesterol rafts diffuse as small entities in the plasma membrane of mammalian cells.. J Cell Biol.

[68] Gaibelet G, Planchenault T, Mazeres S, Dumas F, Arenzana-Seisdedos F (2006). CD4 and CCR5 constitutively interact at the plasma membrane of living cells: a confocal fluorescence resonance energy transfer-based approach.. J Biol Chem.

[69] Funakoshi T, Tachibana I, Hoshida Y, Kimura H, Takeda Y (2003). Expression of tetraspanins in human lung cancer cells: frequent downregulation of CD9 and its contribution to cell motility in small cell lung cancer.. Oncogene.

[70] Mittelbrunn M, Yanez-Mo M, Sancho D, Ursa A, Sanchez-Madrid F (2002). Cutting edge: dynamic redistribution of tetraspanin CD81 at the central zone of the immune synapse in both T lymphocytes and APC.. J Immunol.

[71] Delaguillaumie A, Lagaudriere-Gesbert C, Popoff MR, Conjeaud H (2002). Rho GTPases link cytoskeletal rearrangements and activation processes induced via the tetraspanin CD82 in T lymphocytes.. J Cell Sci.

[72] Varnai P, Bondeva T, Tamas P, Toth B, Buday L (2005). Selective cellular effects of overexpressed pleckstrin-homology domains that recognize PtdIns(3,4,5)P3 suggest their interaction with protein binding partners.. J Cell Sci.

