# Supplementary material for: Preferential Transfer of Certain Plasma Membrane Proteins onto T and B Cells by Trogocytosis
Source: PLoS One. 2010 Jan 14;5(1):e8716. doi: 10.1371/journal.pone.0008716 (PMC2806835; doi:10.1371/journal.pone.0008716)
Supplement: Comment S1 — (0.05 MB DOC) [file pone.0008716.s001.doc]

**Comment S1**

**Comment to Figure S2.** For these experiments, we analyzed the variations in transfer efficiency with the expression level of a given protein. For this, we transiently transfected HEK –FcγRII cells with various amounts of vector encoding FcRγ-GFP leading to different levels of protein expression. We then analyzed the transfer efficiency of the FcR γ-GFP protein onto OT-I T cells as a function of the initial expression of the protein on target cells. We found that transfer efficiency gradually increased with the level of expression up to a plateau above which no additional increase was observed (Figure S2A and B). Similar results were also obtained using stable clones of HEK cells expressing various levels of the FcRγ-GFP protein, and subsequently transiently transfected with the vector encoding FcγRII before using those in trogocytosis experiments (not shown). Finally, similar results were also obtained with three different other proteins either efficiently transferred (CD9) or poorly transferred (CXCR4 and CCR5) (Figure S2B). Altogether, these results indicate that over-expression of GFP-tagged proteins, as routinely achieved in our experimental conditions, leads to saturation of capture efficiency by T cells during trogocytosis.
