# Supplementary material for: Preferential Transfer of Certain Plasma Membrane Proteins onto T and B Cells by Trogocytosis
Source: PLoS One. 2010 Jan 14;5(1):e8716. doi: 10.1371/journal.pone.0008716 (PMC2806835; doi:10.1371/journal.pone.0008716)
Supplement: Comment S2 — (0.03 MB DOC) [file pone.0008716.s002.doc]

**Comment S2.**

**Comment to Figure S3.** Since our results show that proteins transferred during trogocytosis were all present at the PM, we analyzed by confocal microscopy if the proportion of these proteins present at the PM versus intracellular compartments could be higher for proteins efficiently transferred than for proteins poorly transferred. To evaluate PM labelling, we performed MHC class I staining of HEK-FcγRII transfected with FcRγ-GFP. This staining was used to create a mask corresponding to the PM, which was then used to distinguish GFP signals overlapping with the mask (GFP at the PM) from those not overlapping (GFP in intracellular compartments (Figure S3A). By analyzing various Z-sections of an HEK clone stably expressing-GFP, we found that the ratio of PM versus intracellular GFP was only moderately affected along the Z-axis (Figure S3B). Using four different clones of HEK cells expressing different levels of FcRγ-GFP, we found that the higher FcRγ-GFP, the lower the ratio of PM versus intracellular GFP. Thus the ratio is altered by protein expression such that high levels of expression lead to saturation of signal at the membrane and accumulation in the cytoplasm (Figure S3C). This provides a potential explanation for the observation that, beyond a certain threshold, further increase in the level of expression of the protein fused to GFP in the target cell does not result in increased amounts of that protein being captured by trogocytosis.

When we measured the ratio for four other proteins, two being transferred efficiently and two poorly, we found no significant differences in the ratio between PM and intracellular staining with those four proteins (Figure S3D), suggesting that there is no apparent correlation between the fraction of a given protein present at the PM and its transfer efficiency by trogocytosis. All in all, these experiments strongly support the notion that quantitative differences among the transfer efficiency of various proteins present at the PM by T cells reflect selectivity in transfer.
