# Supplementary figures and images for: Preferential Transfer of Certain Plasma Membrane Proteins onto T and B Cells by Trogocytosis
Source: PLoS One. 2010 Jan 14;5(1):e8716. doi: 10.1371/journal.pone.0008716 (PMC2806835; doi:10.1371/journal.pone.0008716)

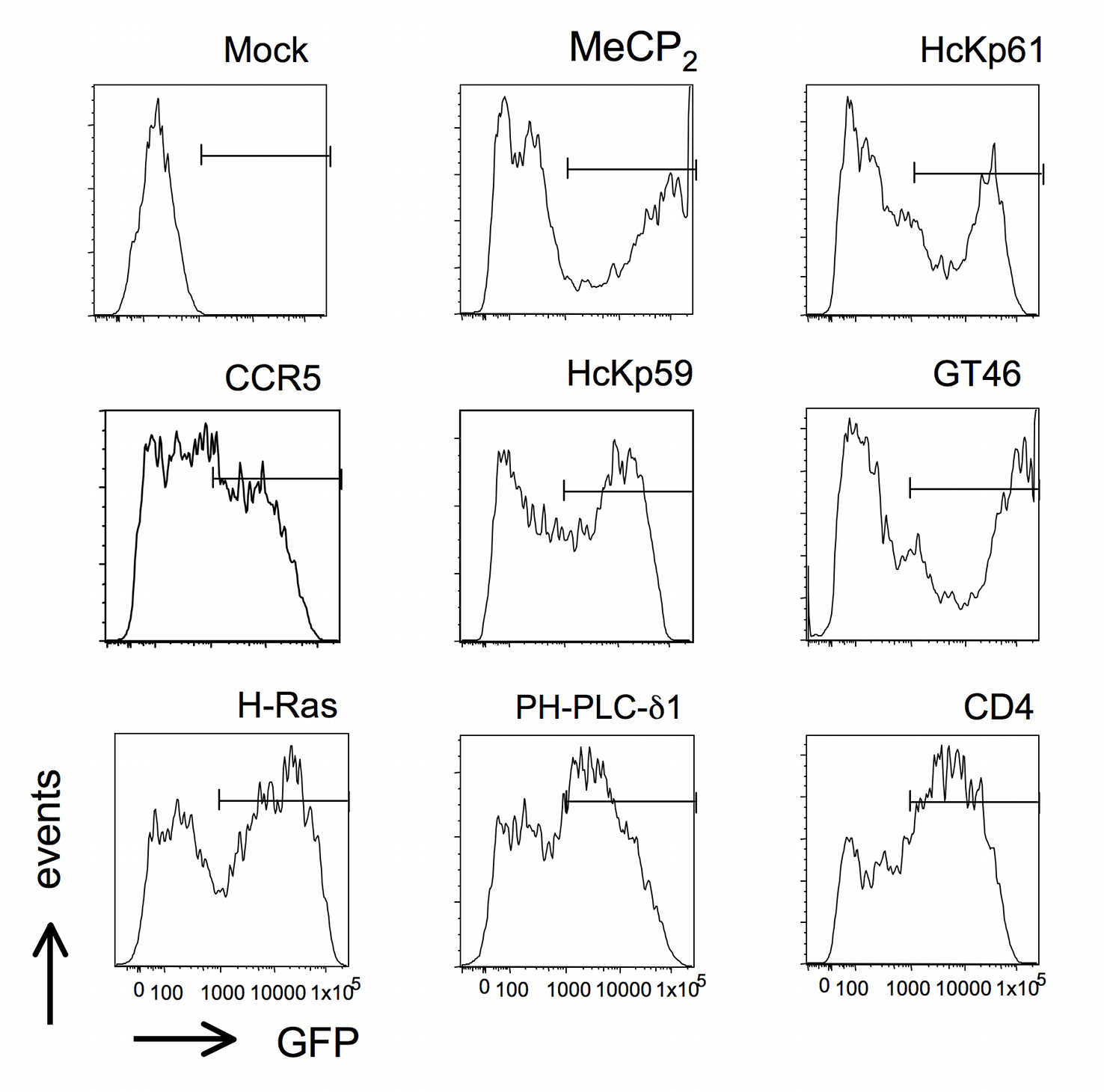

Supplement: Figure S1 — Examples of the levels of expression attained after transient transfection with plasmids coding for various proteins fused to GFP. Typical examples of flow cytometry analyses 48 hours after transient transfection in HEK-FcγRII of 9 different proteins fused to GFP. This type of analysis was performed systematically for all proteins used in this study to ensure that strong expression of the GFP-tagged proteins was detected in at least 40% of HEK-FcγRII. (0.81 MB TIF) [file pone.0008716.s003.tif]

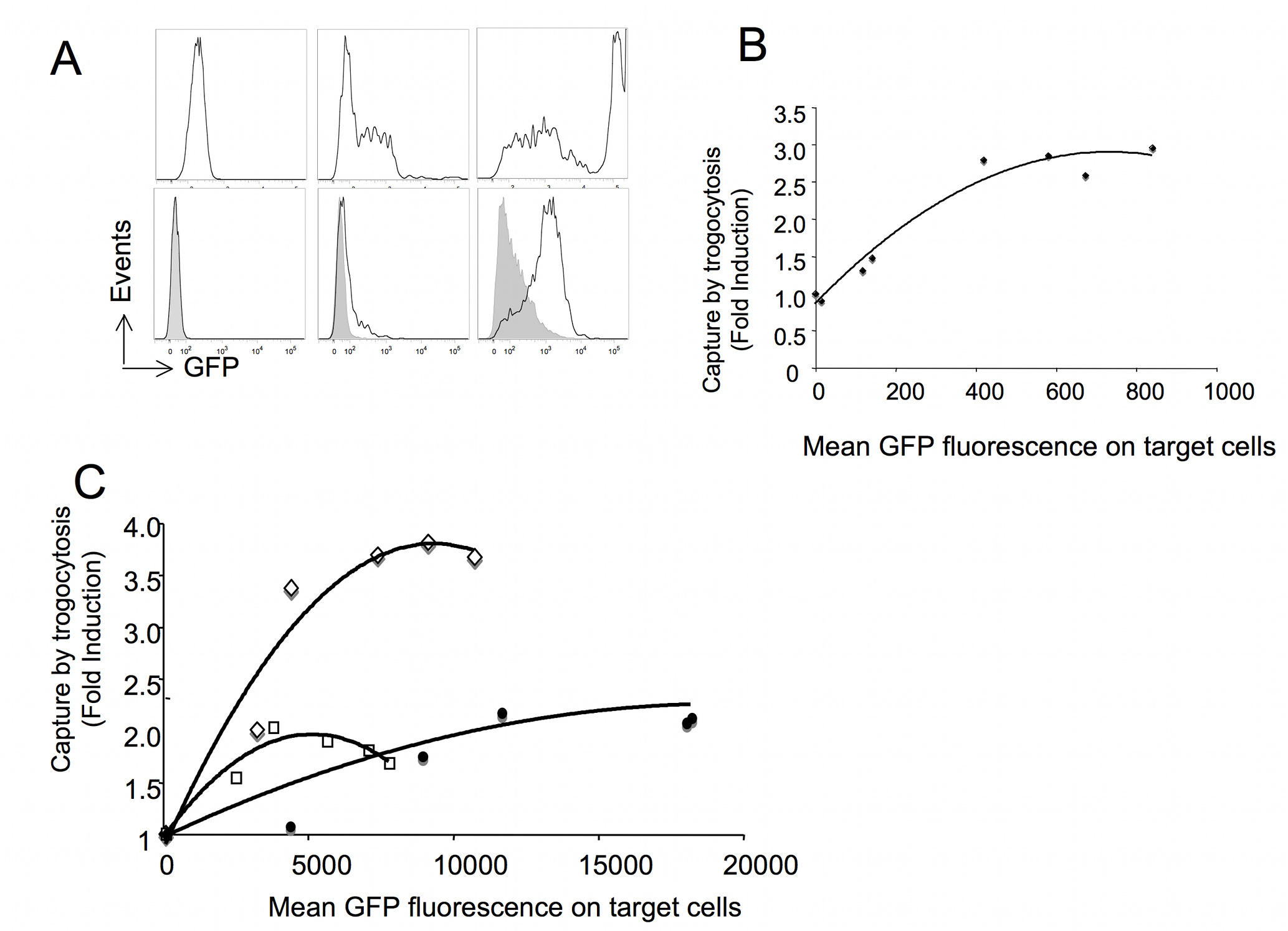

Supplement: Figure S2 — The transfer efficiency of a given protein by trogocytosis is not directly related to its level of expression by target cells. A) Expression by HEK-FcγRII cell of the FcRγ-GFP protein expressed after transient transfection with increasing amounts of vector encoding FcRγ-GFP is shown in the top panels. Capture of the FcRγ-GFP by gated OT-I cells exposed to the target cells shown in top panels in the presence (white histograms) or absence (grey histograms) of the Y3 mAb. B) The graph shows the fold induction of FcRγ-GFP capture by OT-I cells as a function of FcRγ-GFP expression on target cells, compiled from eight separate transfections attaining different levels of FcRγ-GFP expression. C) As in B) except that various amounts of vector DNA coding for CD9 (empty diamonds), CXCR4 (empty squares) or CCR5 (full circles) were used to transfect HEK-FcγRII cells. (1.02 MB TIF) [file pone.0008716.s004.tif]

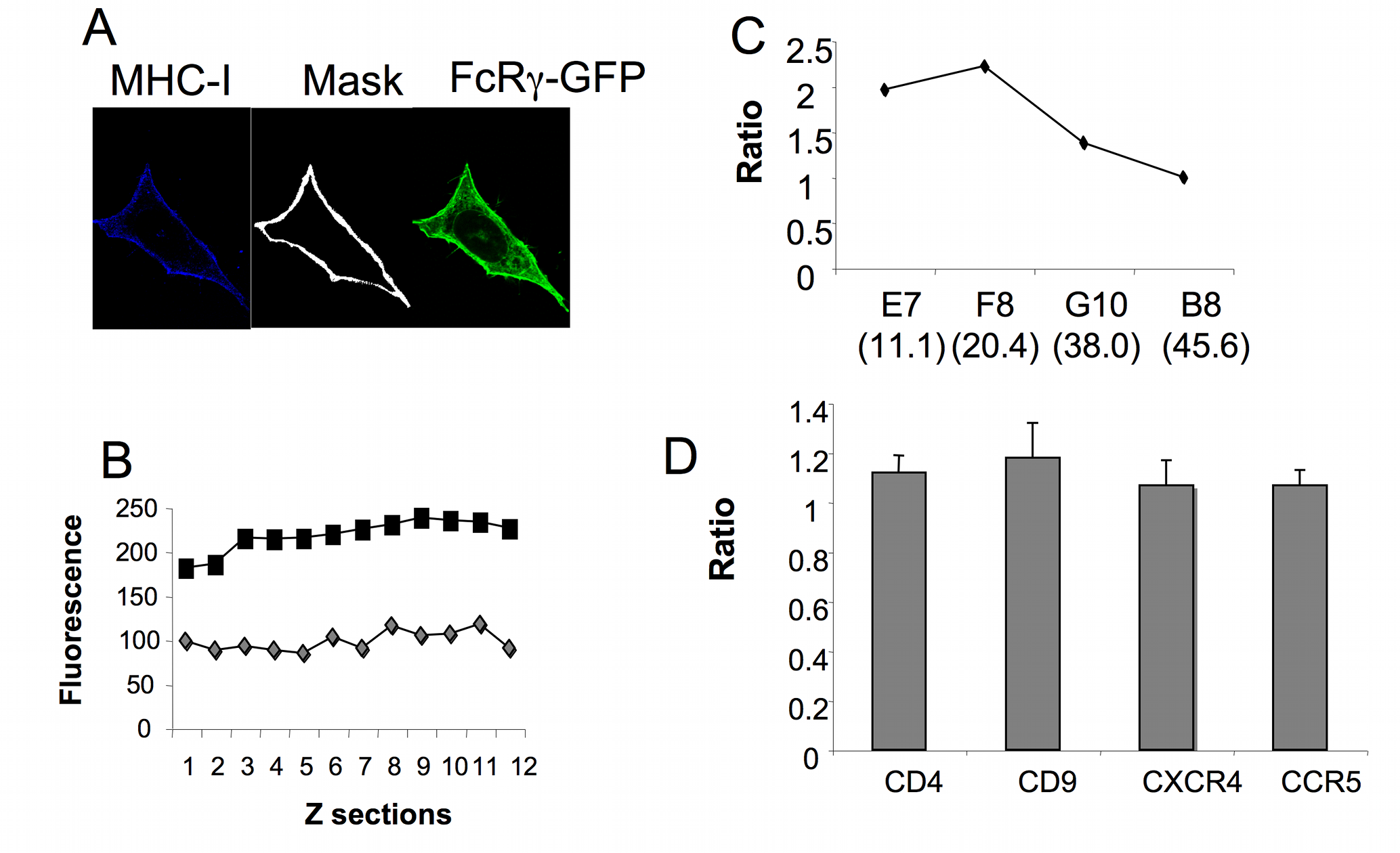

Supplement: Figure S3 — Differences in the transfer efficiency of various GFP proteins on T or B cells are not correlated to their expression levels at the PM. A) A mask (middle panel) delimitating the PM of HEK-FcRγGFP was constructed using the Metamorph software based on extra-cellular anti-MHC class I staining (left panel) and was applied on FcRγ-GFP staining in order to discriminate between GFP fluorescence present at the membrane and intracellularly. B) Arbitrary fluorescent units given by the software on FcR γ-GFP present at the PM (squares) or intracellularly (diamonds) are given for 12 different slices of transfected cells (0.5µm difference between each slice). C) The ratio between cell surface versus intracellular GFP fluorescence is shown for 4 different clones of HEK-expressing increasing levels of FcRγ-GFP (numbers below the clone names refer to the ratio of the mean fluorescence intensity of total FcRγ-GFP expression for each clone divided by that of untransfected HEK cells, measured by flow cytometry). D) The ratio between cell surface versus intracellular GFP fluorescence was similarly calculated on HEK cells transfected with four different vectors encoding either CD4, CD9, CXCR5 or CCR5 fused to GFP. CD4 and CD9 were chosen as examples of proteins efficiently transferred during trogocytosis and CXCR5 and CCR5 as examples of proteins poorly transferred. (0.90 MB TIF) [file pone.0008716.s005.tif]
